# SARS-CoV-2 inhibition and specific targeting of infected cells by VSV particles carrying the ACE2 receptor

**DOI:** 10.1038/s41392-023-01492-7

**Published:** 2023-05-21

**Authors:** Fabian Zech, Stefanie Weber, Hanna Dietenberger, Linyun Zhang, Sabrina Noettger, Meta Volcic, Tim Bergner, Clarissa Read, Konstantin M. J. Sparrer, Thomas F. E. Barth, Frank Kirchhoff

**Affiliations:** 1 Institute of Molecular Virology, Ulm University Medical Center, Ulm, Germany; 2 Institute of Pathology, Ulm University, Ulm, Germany; 3 Central Facility for Electron Microscopy, Ulm University, Ulm, Germany

**Keywords:** Translational research, Molecular medicine

**Dear Editor**

Effective vaccines and some antiviral agents against SARS-CoV-2, the causative agent of coronavirus disease 2019 (COVID-19), have been developed. Nonetheless, additional therapeutics are needed since emerging SARS-CoV-2 variants evade immunity induced by vaccination or previous infection and are resistant to therapeutic antibodies and fusion inhibitors.^1^ In addition, many patients experience prolonged post-acute sequelae of SARS-CoV-2, also known as Long COVID.^2^ One reason for this is the prolonged presence of SARS-CoV-2 in various anatomic sites for several months.^3^ In addition, impaired immune function due to advanced age, immunosuppressive therapy, autoimmune diseases, and comorbidities, such as diabetes or heart disease, as well as effective immune evasion of emerging SARS-CoV-2 variants may delay viral clearance.^4,5^ Thus, the elimination of SARS-CoV-2 infected cells is of great interest to prevent severe COVID-19 and to reduce the risk of long-term complications.^6^ However, none of the available antiviral agents target SARS-CoV-2-infected cells.

Vesicular stomatitis virus (VSV) particles pseudo-typed with the SARS-CoV-2 Spike instead of the VSV-G glycoprotein are commonly used to study SARS-CoV-2 entry and neutralization. The primary receptor of SARS-CoV-2 is Angiotensin-converting enzyme 2 (ACE2), a single-pass type I membrane protein of ~805 amino acids.^7^ Here, we examined whether reversing this system, i.e. pseudo-typing VSV with ACE2, allows specific targeting and inhibition of both SARS-CoV-2 particles and infected cells. VSV replicates very fast and usually kills infected cells within less than a day by causing cytopathic effects and apoptosis. Thus, VSV is applied in oncolytic virus therapy to eliminate cancer cells.^8^ Therefore, we hypothesized that VSV particles carrying ACE2 may not only inactivate SARS-CoV-2 particles but also allow specific infection and killing of Spike-expressing SARS-CoV-2 infected cells (Fig. 1a). To produce pseudo-typed viral particles, HEK293T cells were transfected with ACE2 or Spike (for control) expression vectors (Supplementary Fig. 1) and infected with VSVΔG(GFP) one day later. Viral pseudo-particles (pp) in the supernatant of the producer cells were concentrated by high-speed ultracentrifugation (Supplementary Fig. 2) and ACE2 was readily detectable in both the cellular extracts and the virion-containing cell culture supernatants (Fig. 1b). To determine whether VSVΔG(GFP)ACE2pp infect cells in a Spike-dependent manner, HEK293T cells that were left untreated or transfected with Spike expression construct were incubated with different dilutions of virus stocks. Automated image-based quantification of GFP+ cells revealed that VSVΔG(GFP)ACE2pp efficiently infected Spike-expressing HEK293T cells but not the control cells (Fig. 1c). Flow cytometric and live cell imaging analyses confirmed that VSVΔG(GFP)ACE2pp exclusively targeted the subpopulation of HEK293T cells expressing the SARS-CoV-2 Spike (Fig. 1d, Supplementary Fig. 3 and Movies 1 and 2). Altogether, these results demonstrated that ACE2 is efficiently incorporated into VSV particles and allows selective infection of Spike-expressing target cells.

**Fig. 1:**
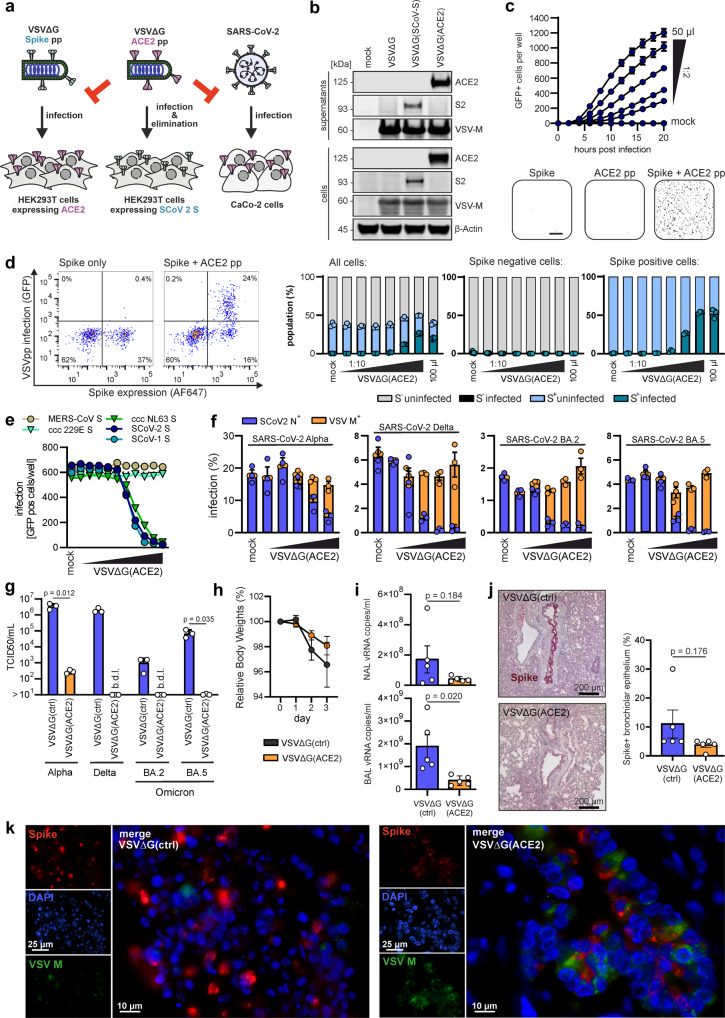
Inhibition of SARS-CoV-2 and specific targeting of Spike-expressing cells by VSVΔG-ACE2 pseudo-particles. **a** Schematic representation of a VSVΔG-ACE2 particle, selectively infecting SARS-CoV-2 Spike^+^ cells, while blocking Spike-mediated infection of pseudo-typed and wt virus. **b** Exemplary immunoblots of whole cells lysates (WCLs) and VSVpp containing supernatants of HEK293T cells transfected with vectors expressing the SARS-CoV-2 Spike or ACE2 proteins and infected with VSVΔG-GFP. Blots were stained with anti-ACE2, anti-Spike, anti-ß-actin, and anti-VSV-M antibodies. **c**, Infection kinetics of Spike^+^ HEK293T cells inoculated with the indicated volumes of VSVΔG-ACE2. Infected GFP^+^ cells were automatically quantified over a period of 20 h (upper panel). Binary images of HEK293T cells transduced with VSVΔG-ACE2, transfected with SARS-CoV-2 Spike, or both (lower panel). Infection events (i.e. GFP+ cells) are displayed as black dots. **d** Exemplary gating to detect Spike expressing and VSVΔG infected HEK293T cells (left panel). FACS analysis of Spike expressing HEK293T cells infected with increasing amounts of VSVΔG-ACE2 (right panel). Shown are the percentages of VSVΔG-ACE2 infected cells in the total, Spike-negative and Spike-positive cell population. **e** Infection of Caco-2 cells with VSVΔG-GFP pseudotyped with the indicated coronavirus Spike proteins. Spike-carrying VSVpp were pre-treated (30 min, 37 °C) with VSVΔG-ACE2 in the ratios 1:3, 1:2 and 1:1 to 1:10^−7^ in 10-fold dilution steps. **f** Inhibition of genuine SARS-CoV-2 strains by VSVΔG-ACE2. Caco-2 cells were inoculated with SARS-CoV-2 NL-02-2020, Delta and Omicron (MOI 0.05) variants pre-treated with VSVΔG-ACE2 at the ratios 1:3, 1:2, 1:1 and 1:0.1. Two days after infection, the cells were harvested, stained with anti-Spike Ab and analysed by FACS. **g** Infectious SARS-CoV-2 particles in the supernatants of Caco-2 cells infected with mock or VSVΔG(ACE2)pp-treated SARS-CoV-2. The samples correspond to the cultures treated with naked control VSVΔG particles or the highest concentration of VSVΔG(ACE2)pp treated cultures shown in panel f. TCID_50_ was determined by infection of Caco-2 cells as described in the methods section. b.d.l, below detection limit. (*n* = 3, ± SEM; p-values were determined using a two-tailed Student’s t test with Welch’s correction. **h**, Relative body weights of Syrian gold hamsters following infection with SARS-CoV-2 Delta and administration of control or VSVΔG-ACE2. **i**, Viral RNA loads in nasal and bronchoalveolar lavages (NAL and BAL) of control or VSVΔG-ACE2 treated hamsters three days post-infection with the SARS-CoV-2 Delta variant. **j**, Lung histology of SARS-CoV-2 delta infected Syrian gold hamsters treated with control or VSVΔG-ACE2. SARS-CoV-2 Spike (red) and hematoxylin (blue) (left panel) and quantification of the corresponding Spike expression (right panel). **k** Fluorescence immunohistology of the lung of SARS-CoV-2 Delta infected Syrian gold hamsters treated with control (left panel) or VSVΔG-ACE2 (right panel) and stained for Spike and VSV M protein expression

To examine whether VSVΔG-ACE2pp inhibit ACE2-mediated virus infection, we generated VSVΔG(GFP)pp carrying the Spike proteins of five different human coronaviruses. Pre-exposure to increasing numbers of VSVΔG-ACE2pp inhibited infection by viral pseudo-particles carrying the SARS-CoV-1, SARS-CoV-2 and hCoV-NL63 Spike proteins that utilize ACE2 as entry receptor. In contrast, VSVΔG-ACE2pp had no effect on infection mediated by the MERS-CoV and hCoV-229E Spike proteins that do not utilize ACE2 (Fig. 1e). VSVΔG-ACE2pp obtained before and after ultracentrifugation showed similar inhibitory activity (Supplementary Fig. 4a). Thus, VSVΔG-ACE2 particles specifically block Spike proteins of ACE2-tropic coronaviruses. Control experiments showed that they do not cause cytotoxic effects in cultures lacking Spike expression, are stable for ~2 days at room temperature, and inhibit Spike-mediated infection about as efficiently as soluble ACE2 (Supplementary Fig. 4b–d).

To determine whether VSVΔG(GFP)ACE2 particles inhibit genuine SARS-CoV-2, we used Caco-2 cells that are highly susceptible to infection. Electron microscopy verified that the bullet-shaped VSVΔG-ACE2pp and spherical SARS-CoV-2 particles frequently clustered together, indicating the interaction between the ACE2 and Spike proteins (Supplementary Fig. 5). Pre-treatment of the SARS-CoV-2 Alpha, Delta and Omicron BA.2 and BA.5 variants of concern with VSV-ACE2pp dose-dependently reduced SARS-CoV-2 nucleocapsid (N) positive cells (Fig. 1f). At higher doses, most N+ cells also stained positive for the VSV matrix (M) protein. Treatment with VSVΔG(GFP)ACE2pp prevented the production of infectious SARS-CoV-2 particles (Fig. 1g). Thus, VSVΔG-ACE2pp allowed to super-infect the great majority of Spike-expressing SARS-CoV-2 infected cells and to prevent infectious virus production.

To assess the protective effects of VSVΔG-ACE2pp in vivo, we used the Syrian gold hamster model that is well-established for studies on SARS-CoV-2 infection and the evaluation of antiviral drugs.^9^ Ten animals were infected with SARS-CoV-2 Delta (B.1.617.2; 2 × 10^4^ PFU) via intranasal administration and intranasally treated with naked (*n* = 5, group 1) or ACE2 pseudo-typed (*n* = 5, group 2) VSVΔG particles 4 h before and after virus exposure allowing targeting of both SARS-CoV-2 particles and infected cells. Health status, body weights and temperatures were monitored until the animals were euthanized at day 3 postinfection. Animals in both groups developed symptoms associated with SARS-CoV-2 infection, including fatigue and cough, as well as a loss of body weight due to decreased appetite and increased energy expenditure caused by the infection. On average, animals treated with VSVΔG-ACE2pp lost ~ 1.9% of their body weight compared to ~3.4% in the control group (Fig. 1h). SARS-CoV-2 Delta replicated with high efficiency in the lungs of Syrian hamsters reaching average viral RNA loads of ~ 1.6 × 10^8^ and ~ 1.8 × 10^9^ copies per ml in nasal and bronchoalveolar lavage, respectively (Fig. 1i). On average, VSVΔG-ACE2pp treatment reduced viral RNA loads by ~5-fold. In the nasal mucosa, subepithelial round cell infiltrates with some intermingled neutrophilic granulocytes were detected leading to an erosive disruption of epithelial cells (Supplementary Fig. 6a, b). Spike was detected in a sector-like fashion in the ciliated cells (Supplementary Fig. 6c, d). In the lung parenchyma, the round cell infiltrate was limited to the bronchioles, while the alveolar ducts and alveoli showed no infiltrates (Supplementary Fig. 7a, b). Immunohistochemistry showed sector-like Spike stains of epithelial cells lining the bronchioles in the control group. On average, the number of Spike+ cells was reduced in VSV-ACE2pp treated animals (Fig. 1j, Supplementary Fig. 7c, d). Importantly, most SARS-CoV-2 infected Spike+ cells in the treatment group also stained positive for the VSV M protein indicating targeting by VSVΔG-ACE2pp in vivo (Fig. 1k). Altogether, the results show that VSVΔG-ACE2pp inhibit SARS-CoV-2 and diminish the severity of SARS-CoV-2 infection in the hamster model.

In summary, we demonstrate that ACE2 is efficiently incorporated into VSV particles and inhibits infection by ACE2-tropic human coronaviruses. In addition, VSVΔG-ACE2pp specifically target and infect Spike-expressing cells and prevent infectious SARS-CoV-2 production. Thus, unlike other therapeutics, they do not only inhibit the virus but should also allow selective elimination of SARS-CoV-2 infected Spike-expressing cells. While our approach might be useful for early treatment to prevent viral spread and allow fast recovery with mild symptoms, it should be particularly beneficial for the treatment of individuals who do not clear the virus even months after primary SARS-CoV-2 infection. Since ACE2 is the essential entry factor for all SARS-CoV-2 variants, the development of resistance is highly unlikely. VSV replicates with high efficiency and large numbers of VSVΔG-ACE2 particles could be used to purge SARS-CoV-2 out of individuals showing evidence for long-term and severe COVID-19. Even long-term treatment should be well-tolerated because VSVΔG-ACE2 particles can only enter cells that express the Spike protein and are hence already infected by SARS-CoV-2. Our proof-of-concept evidence shows the safety and efficacy of VSVΔG-ACE2pp both in cell culture and in vivo in the Syrian hamster model. The in vivo efficacy of our approach can most likely be improved by utilizing recombinant replication-competent VSVΔG-ACE2 constructs, which may spread until SARS-CoV-2 infected cells are eliminated. Altogether, our results introduce a novel therapeutic approach and suggest that VSVΔG-ACE2 particles targeting both SARS-CoV-2 virions, as well as infected cells in a highly selective manner may be safe and effective.

## Supplementary information

Supplementary Materials

Infection of Spike-expressing cells by VSVΔG-ACE2 particles example 1

Infection of Spike-expressing cells by VSVΔG-ACE2 particles example 2

## Data Availability

All data are available from the corresponding authors. Raw and analyzed data is available through the Mendeley Data Repository 10.17632/x3jyrtnvjj.1.
