# Supplementary material for: SARS-CoV-2 inhibition and specific targeting of infected cells by VSV particles carrying the ACE2 receptor
Source: Signal Transduct Target Ther. 2023 May 21;8:208. doi: 10.1038/s41392-023-01492-7 (PMC10200011; doi:10.1038/s41392-023-01492-7)
Supplement: Supplementary file 1 — Supplementary Materials [file 41392_2023_1492_MOESM1_ESM.docx]

Supplementary Materials for

SARS-CoV-2 inhibition and specific targeting of infected cells by VSV particles carrying the ACE2 receptor

Fabian Zech^1^, Stefanie Weber^1^, Hanna Dietenberger^2^, Linyun Zhang^2^, Sabrina Noettger^1^, Meta Volcic^1^, Tim Bergner^3^, Clarissa Read^3^, Konstantin M.J. Sparrer^1^, Thomas F.E. Barth^2^ & Frank Kirchhoff^1^

^1^Institute of Molecular Virology, Ulm University Medical Center, Ulm, Germany;

^2^Institute of Pathology, Ulm University, Ulm, Germany;

^3^Central Facility for Electron Microscopy, Ulm University, Ulm, Germany.

Correspondence to:

Fabian Zech ([fabian.zech@uni-ulm.de](mailto:fabian.zech@uni-ulm.de)) or Frank Kirchhoff ([frank.kirchhoff@uni-ulm.de](mailto:frank.kirchhoff@uni-ulm.de))

**This PDF file includes:**

1. Materials and Methods
2. Supplementary figures S1 to S7
3. Captions for Movies S1 and S2
4. Supplementary References

1. Materials and Methods

Cell culture and viruses

All cells were cultured at 37°C in a 5% CO_2_ atmosphere. Human embryonic kidney 293T cells purchased from American-type culture collection (ATCC: #CRL3216) were cultivated in Dulbecco’s Modified Eagle Medium (DMEM, Gibco) supplemented with 10% (v/v) heat-inactivated fetal bovine serum (FBS, Gibco), 2 mM L-glutamine (PANBiotech), 100 µg/ml streptomycin (PANBiotech) and 100 U/ml penicillin (PANBiotech). Caco-2 (human epithelial colorectal adenocarcinoma, kindly provided by Prof. Holger Barth, Ulm University) cells were cultivated in DMEM (Gibco) containing 10% FBS (Gibco), 2 mM glutamine (PANBiotech), 100 µg/ml streptomycin (PANBiotech), 100 U/ml penicillin (PANBiotech), 1 mM Non-essential amino acids (NEAA, Gibco), 1 mM sodium pyruvate (Gibco). I1-Hybridoma cells were purchased from ATCC (#CRL-2700) and cultured in RPMI supplemented with 10% (v/v) heat-inactivated FBS (Gibco), 2 mM L-glutamine (PANBiotech), 100 µg/ml streptomycin (PANBiotech) and 100 U/ml penicillin (PANBiotech).

Viruses and SARS-CoV-2 variants

VSVΔG(VSVG)pp were kindly provided by Prof. Karl-Klaus Conzelmann (Gene Center, LMU Munich). The BetaCoV/Netherlands/01/NL/2020 (NL-02-2020, Alpha) lineage were obtained from the European Virus Archive. The SARS-CoV-2 variant B.1.617.2 (Delta) was kindly provided by Prof. Dr. Florian Schmidt and Dr. Bianca Schulte (University of Bonn). The SARS-CoV-2 omicron BA.2 variant (Cat, #NR-56520) and the SARS-CoV-2 omicron BA.5 (Cat, #NR-56798), Isolate hCoV-19/South Africa/CERI-KRISP-K040013/2022 were obtained through BEI Resources.SARS-CoV-2 strains were propagated on Vero E6 (NL-02-2020, Delta), or Calu-3 (Omicron BA.2 and BA.5) cells. 70-90% confluent cells in 10 cm cell culture dishes were inoculated with the SARS-CoV-2 isolate (multiplicity of infection (MOI) of 0.03-0.1) in 3.5 ml serum-free medium. The cells were incubated for 2h at 37°C, before adding 20 ml medium containing 15 mM HEPES (Carl Roth, Cat#6763.1). Virus stocks were harvested as soon as strong cytopathic effect (CPE) became apparent, typically after about 12 hrs. The virus stocks were centrifuged for 5 min at 1,000 g to remove cellular debris, aliquoted, and stored at -80°C until further use.

Expression constructs

HCoV S protein encoding plasmids hCoV-229E-S and hCoV-NL63-S were synthesized by Baseclear, PCR amplified and subcloned into a pCG-IRES_eGFP expression construct using the restriction enzymes XbaI and MluI (New England Biolabs). pCG_MERS-CoV-S-V5_IRES_eGFP and pCG_SARS-CoV-1-S-V5_IRES_eGFP were provided by Michael Schindler (Tübingen University) pCG_SARS-CoV-2-S-V5-IRES_eGFP was generated as previously described^1^.

Tissue Culture Infection Dose50 (TCID50) endpoint titration

SARS-CoV-2 stocks or infectious supernatants were serially diluted on Caco-2 (15,000), seeded in 96 F-bottom plates in 100 µl medium and incubated overnight. 100 µl of diluted SARS-CoV-2 stocks or infectious supernatants were used for infection, resulting in final dilutions of 1:10^1 to 1:10^12 on the cells in 9 technical replicates. Cells were incubated for 7 days and monitored for CPE. TCID50/ml was calculated according to the Reed and Muench method.

Pseudo and decoy particle production

To produce pseudotyped VSVΔG-GFP particles, 6*10^6^ HEK 293 T cells were seeded 18 h before transfection in 10 cm dishes. The cells were transfected with 15 µg of a glycoprotein expressing or ACE2 vector using Polyethylenimine (PEI, 1 mg/ml in H_2_O, Sigma-Aldrich). 24 h post-transfection, the cells were infected with VSVΔG-GFP particles pseudotyped with VSV G at an MOI of 3. One h post-infection, the inoculum was removed. Pseudotyped VSVΔG-GFP particles were harvested 16 h post-infection. Cell debris were pelleted and removed by centrifugation (500 g, 4 °C, 5 min). Residual input particles carrying VSV-G were blocked by adding 10 % (v/v) of I1 Hybridoma supernatant (I1, mouse hybridoma supernatant from CRL-2700; ATCC) to the cell culture supernatant.

Transfections

Plasmids encoding for the respective coronavirus Spike protein or human ACE2 (Suppementary Fig. 1) were transfected using either calcium phosphate transfection or Polyethylenimine according to the manufacturer's recommendations or as described previously^2^.

Whole-cell and cell-free lysates

Whole-cell lysates were prepared by collecting cells in Phosphate-Buffered Saline (PBS, Gibco), pelleting (500 g, 4 °C, 5 min), lysing and clearing as previously described^2^. The total protein concentration of the cleared lysates was measured using the Pierce BCA Protein Assay Kit (Thermo Scientific) according to manufacturer’s instructions. Viral particles were filtered through a 0.45 µm MF-Millipore Filter (Millex) and centrifuged through a 20% sucrose (Sigma) cushion. The pellet was lysed in transmembrane lysis buffer already substituted with Protein Sample Loading Buffer (LI-COR).

SDS-PAGE and immunoblotting

SDS-PAGE and immunoblotting were performed as previously described^2^. In brief, whole-cell lysates were separated on NuPAGE 4-12% Bis-Tris Gels (Invitrogen) for 90 min at 120 V and blotted at constant 30 V for 30 min onto Immobilon-FL PVDF membrane (Merck Millipore). After the transfer, the membrane was blocked in 1% Casein in PBS (Thermo Scientific) and stained using primary antibodies directed against SARS-CoV-2 S (1:1,000, Biozol, 1A9, #GTX632604), ACE2 (1:1,000, Abcam, #GTX3344245-4), VSV-M (1:2,000, Absolute Antibody, 23H12, #Ab01404-2.0), GAPDH (1:1,000, BioLegend, #W17079A) and Infrared Dye labelled secondary antibodies IRDye 800CW Goat anti-Mouse #926-32210, IRDye 800CW Goat anti-Rat (#926-32219), IRDye 680CW Goat anti-Rabbit (#925-68071), IRDye 680CW Goat anti-Mouse (#926-68070), IRDye 800CW Goat anti-Rabbit (#926-32211) all 1:10,000. Proteins were detected using an LI-COR Odyssey scanner and band intensities were quantified using LI-COR Image Studio version 5.

qRT-PCR

N (nucleoprotein) RNA levels were determined in supernatants collected from SARS-CoV-2 infected cultures. Total RNA was isolated using the Viral RNA Mini Kit (Qiagen) according to the manufacturer’s instructions. qRT-PCR was performed according to the manufacturer’s instructions using TaqMan Fast Virus 1-Step Master Mix (Thermo Fisher) and a OneStepPlus Real-Time PCR System (96-well format, fast mode). Primers were purchased from Biomers and dissolved in RNAse-free water. Synthetic SARS-CoV-2-RNA (Twist Bioscience) was used as a quantitative standard to obtain viral copy numbers. All reactions were run in duplicates. Forward primer HKU-NF: 5’-TAA TCA GAC AAG GAA CTG ATT A-3’; Reverse primer HKU-NR: 5’-CGA AGG TGT GAC TTC CAT G-3’; Probe HKU-N): 5’-FAM-GCA AAT TGT GCA ATT TGC GG-3’TAMR).

Pseudoparticle inhibition

VSVpp inhibition experiments were performed as previously described^1^. In brief, Caco-2 cells were infected with 100 µl freshly produced VSVΔG-GFP pseudo particles, which were preincubated (30 min, 37°C) with the indicated volume of VSVΔG(ACE-2) particles. GFP-positive cells were automatically quantified using a Cytation 3 microplate reader (BioTek).

Flow cytometry

To determine SARS-CoV-2 infection by flow cytometry, two days post infection, the cells were harvested, washed in PBS with 1% FCS, fixed by adding 1% PFA and permeabilized using the NORD BIO FIX&PERM kit according to the manufacturer’s instructions. Cells were stained using anti-SARS-CoV-2 N (Sino Biological, #40143-R004A) and anti-RB AF647 (Invitrogen, #A32733) antibodies and flow cytometric measurements were performed using a BD FACS Canto II flow cytometer. The Data was analysed with FlowJo Version 10.

Cell Viability

Cell viability of CaCo-2 cells treated with VSVΔG(GFP)ACE2pp was determined using the CellTiter-Glo kit (Promega Corp, Madison, WI) according to the manufacturer’s instructions. In short, 10.000 Caco-2 cells per well were seeded in 96-well format and transduced with VSVΔG(GFP)ACE2pp. 24 h after transduction, 100 µl of CellTiter-Glo® 2.0 reagen was added to the cells and luminescence was quantified using a Cytation 3 microplate reader (BioTek).

VSVΔG(ACE2)pp stability

To assess the stability of VSVΔG(GFP) particles, we incubated the particles at three different temperatures: -80°C, room temperature (RT), and 37°C over a period of 82 hours. At the indicated time points, aliquots of the particles were taken and frozen -80°C. After the last time point all aliquots were tranduced on HEK293T cells expressing SARS-CoV-2 S. GFP-positive cells were automatically quantified 24 h later using a Cytation 3 microplate reader (BioTek)

Histology and immunofluorescence

Organs were fixed in buffered formalin (5% formaldehyde) for 72 hours. Paraffine sections of 2-3µm thickness were stained with Hematoxylin-eosin according to routine protocols. Immunohistochemistry was performed on 2-3µm thick sections of FFPE tissue according to standard protocols^3^. Antigen retrieval was performed by microwave heating in citrate buffer, pH 6, for 20 min. Sections were incubated for 1 hr with anti-Spike Ab (clone HL257, dilution 1:100; Abcam, Cambridge, UK). Bound antibodies were visualized by the DAKO REAL Detection System (alkaline phosphatase/ RED/ Rabbit/Mouse, K5005, Agilent; DAKO, Jena, Germany) and counterstained with hematoxylin. Images were taken with a photo microscope (Axiophot, Oberkochen, Germany) with a Charge-coupled Device (CCD) camera (JVC, KY-F75U, Yokohama, Japan) and the software Diskus (Hilgers Technisches Büro, Königswinter, Germany). Slides were evaluated in a blinded fashion on a multihead microscope by two experienced pathologists. Inflammation was evaluated in hot spots and graded from 0-3 (0= no inflammation; 1= scarce round cell infiltrates, i.e., max. 5 cells/high-powerfield (HPF; 400x magnification)2 = medium infiltrates; i.e., max 10 cells/HPF; heavy infiltrate > 10 cells/HPF). Immunohistochemical stainings were evaluated in a semiquantitative fashion; scores and percentage of positive cells were used for further analysis using GraphPad PRISM 9; Boston, MA, USA). Immunofluorescence stainings were performed as a double stain with SARS-CoV-2 spike antibody (1:100, Biozol, 1A9, #GTX632604) and VSV-M antibody (1:250, Absolute Antibody, 23H12, #Ab01404-2.0). Antigen retrieval was performed in a steamer using citrate buffer pH 6.1. The fluorochrome used for SARS-CoV-2 spike protein labelling was Cy3 conjugated Anti-rabbit IgG (1:800, Dianova, polyclonal, #111-165-144). For VSV-M we used biotinylated Anti-mouse antibody (1:400, Dianova, polyclonal, #115-066-062) as secondary antibody followed by Alexa Fluor 488 Streptavidin (1:1600, Thermo Fisher Scientific, #S11223). The counterstain was performed with DAPI (4′,6-diamidino-2-phenylindole). Negative controls were performed by omitting the primary antibody. Slides were evaluated under a Axiophot microscope (Oberkochen, Germany) coupled to a CCD camera (MetaSytems, Altlussheim, Germany).

Generation of HEK293T cells inducible for Spike expression

Lentiviral particles were produced in HEK293T cells which were transfected with 2.61 µg Gag/Pol, 1.32 µg of Rev, 1.5 µg VSV-G and 5.25 µg of pLVX-TetOne-Puro-CoV2-Spike plasmids. Supernatants were collected 72h post transfection and were used to transduce HEK2239T cells. On a day 4, media was removed, cells were split and seeded into fresh media containing 100 ng/ml of Puromycin. Cells were monitored over the period of 2-3 weeks to select spike stable expressing clones. Expression of Spike was evaluated by addition of 100ng/ml of Doxycyclin into the media, 24h later cells were harvested an western blotting was performed with cell lysates.

Live cell imaging

For the live cell imaging experiment, we used HEK293T cells expressing the Spike protein under doxycycline induction. These cells were mixed with non-induced 293T cells (1:10) to create a heterogeneous population. Spike expression was induced by adding 200 ng/ml doxycycline to the cell culture. After 24 hours, the cells were stained with anti-Spike (Abcam ab273433, 1:1000) primary antibody followed by secondary anti-mouse AF647 (Invitrogen A32728, 1:400) antibody for detection. Cells were washed with DMEM after 2 h and live cell imaging was performed using a Leica LSM 710 confocal microscope. The acquired images were analyzed with ZEN Black Edition software.

Electron Microscopy

Negative staining and transmission electron microscopy was performed as previously described^4^. In brief, 10 µL of the respective supernatants were loaded onto freshly glow-discharged 300 mesh copper grids coated with a carbon reinforced formvar film. After 10 min of adsorption at room temperature, grids were washed three times with aqua bidest and stained with 2% uranyl acetate in aqua bidest. Grids were imaged with a JEOL 1400 transmission electron microscope operated at 120 kV.

Study in Syrian Gold hamsters

The in vivo efficiency study in the hamster model was performed by Scantox Solna, Sweden. Ten Syrian gold hamsters were included in the study, granted by the regional animal ethics committee in Stockholm (2020-2021). Animals were divided into two groups of five, to receive naked VSVΔG particles (control group) or VSVΔG-ACE2 (treatment group) via intranasal administration at 4 h pre-infection and 4 h post-infection. At 0 h, all animals were subjected to infection with SARS-CoV-2 B.1.617.2 (2 x 10^4^ PFU), via intranasal administration. Body weights, health status and body temperature were recorded once daily following infection. On 3 days post-infection (p.i.), a terminal blood sample was collected under anaesthesia, after which animals were euthanised. Broncheoalveolar (BAL) and nasal (NAL) lavage fluid was collected, whereafter lungs and nasal turbinates were excised. Serum was extracted via centrifugation and all tissues and biological fluids were frozen and stored at -80 °C and on dry ice until further analysis.

Statistics

Statistical analyses were performed using GraphPad PRISM 9.2 (GraphPad Software). P-values were determined using a two-tailed Student’s t test with Welch’s correction. Unless otherwise stated, data are shown as the mean of at least three independent experiments ± SEM.

Data Availability

The datasets generated during and/or analyzed during the current study are available from the corresponding authors on request. All [Source data](https://www.nature.com/articles/s41467-021-22655-6#Sec27) are provided in the Supplementary Information/Source Data file.

**2. Supplementary figures**


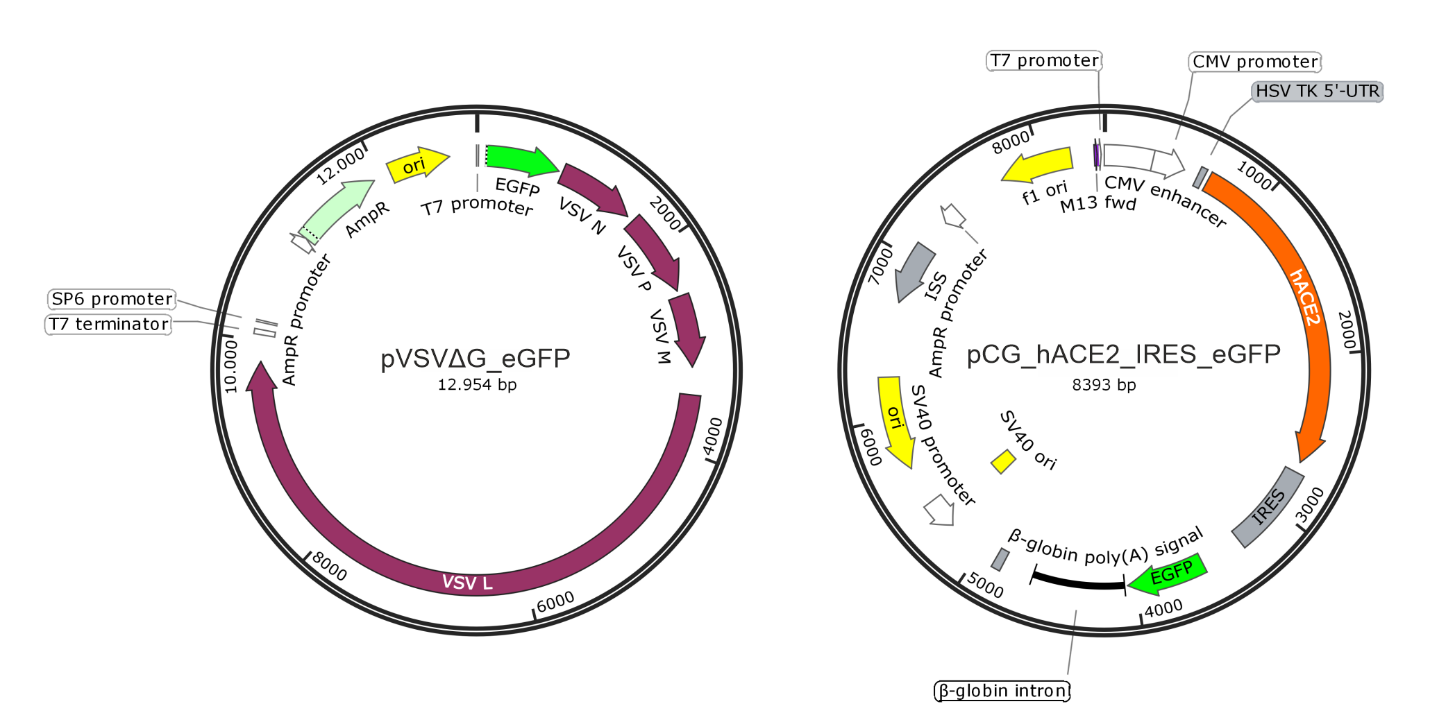


**Supplementary Figure S1: Maps of the plasmids encoding for the VSVΔG backbone and the human ACE2.** The plasmid maps were generated using SnapGene version 5.0.8.

**
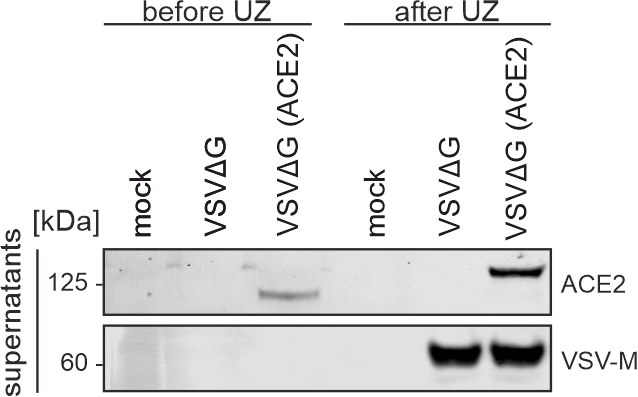
**

**Supplementary Fig. S2: VSVΔG(GFP)ACE2pp are concentrated by high-speed ultra-centrifugation.** Exemplary immunoblots of untreated or Ultracentrifuged supernatant (SN) of HEK293T cells transfected with vectors expressing the ACE2 protein. As indicated, the cells were infected with VSVΔG-GFP and supernatants were either blotted untreated or after concentration using high-speed ultracentrifugation (UZ). Blots were stained with anti-ACE2 and anti-VSV-M antibodies.

**
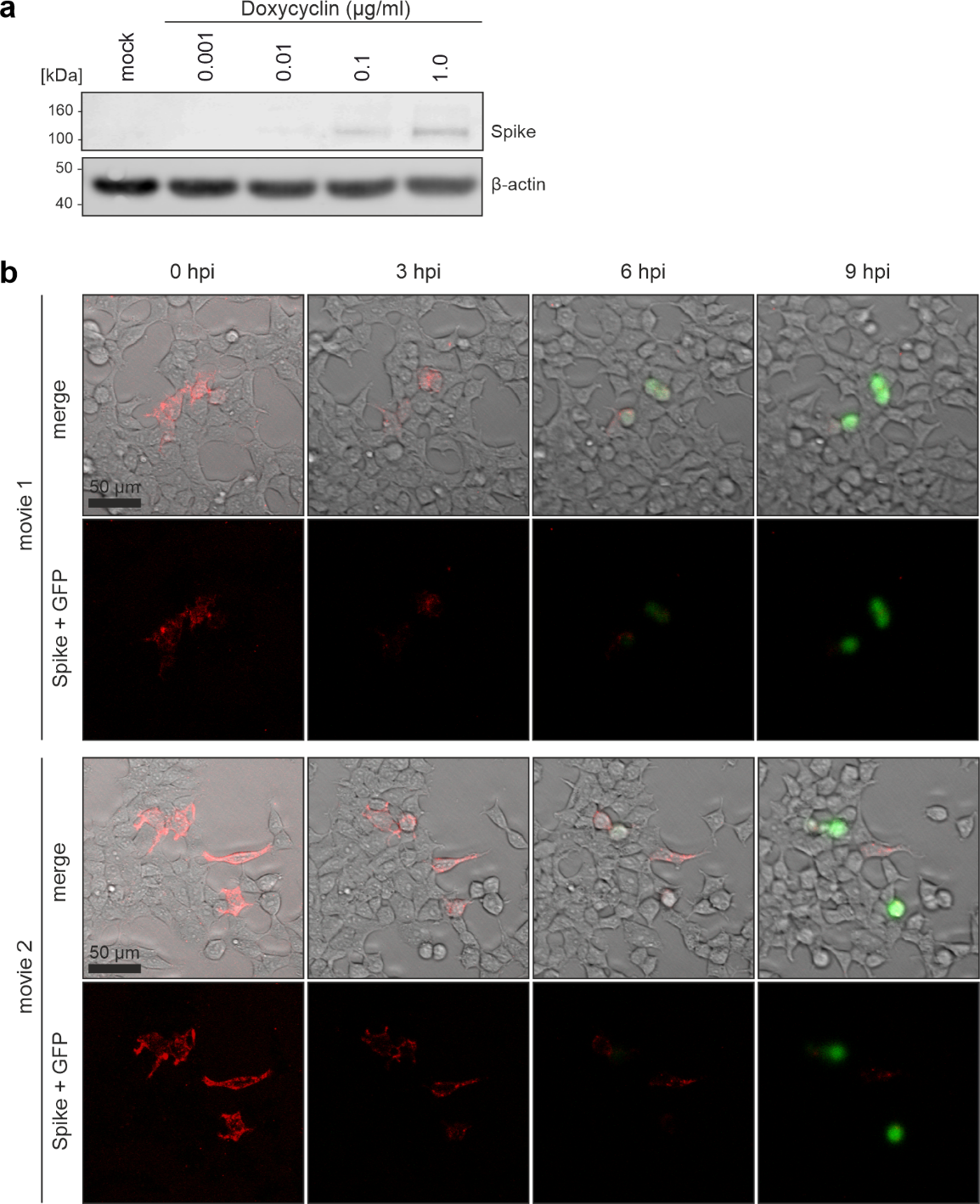
**

**Supplementary Fig. S3: HEK293T cells expressing SARS-CoV-2 Spike are targeted by VSVΔG(ACE2)pp. a,** Immunoblot of whole cell lysates of inducible SARS-CoV-2 Spike expressing HEK293T cells. Spike expression was induced by the indicated amount of Doxycycline. 24 hours after induction, the cells were harvested and immunoblots were performed. Blots were stained for SARS-CoV-2 Spike (1A9) and β-actin. **b,** Exemplary time points of the live cell imaging (Supplemental movies 1 and 2). VSVΔG(ACE2)pp were transferred onto a heterogeneous population of WT and SARS-CoV-2 S-expressing HEK293T cells. The cells were monitored for Spike (red) expression and VSVΔG (GFP) infection over a period of 10 hours in 30-minute intervals.

**
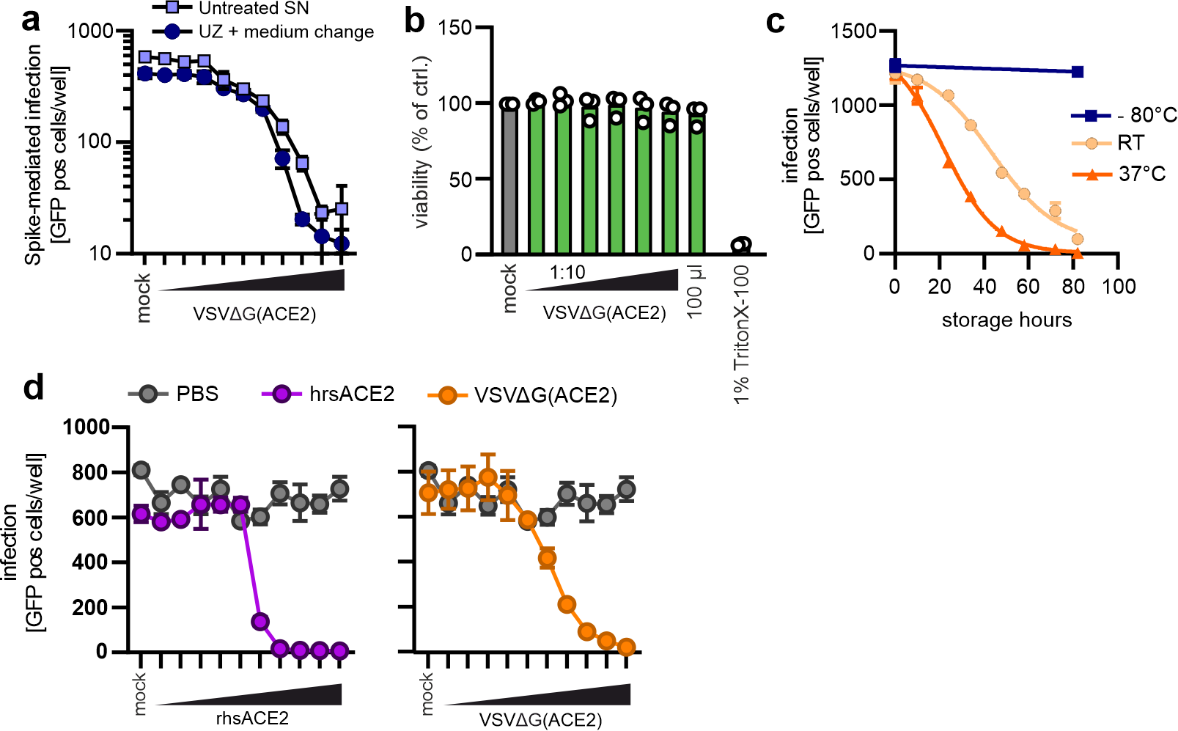
**

**Supplementary Fig. S4: Activity and stability of purified VSVΔG(ACE2)pp. a**, Automated quantification of GFP fluorescence of Caco-2 cells infected with VSVΔG-GFP pseudotyped with the SARS-CoV-2 Spike protein. The Spike carrying VSVpp were pre-treated (30 min, 37°C) with the indicated amounts of untreated or concentrated VSVΔG-ACE2 particles. Lines represent the mean of three independent experiments (±SEM) **b**, Viability of Caco-2 cells after VSVΔG(ACE2) exposure. Caco-2 cells were exposed to the indicated amounts of VSVΔG(ACE2) or 1% Triton X-100 for two days. Cell viability was determined using CellTiter-Glo assay. Lines represent the mean of three independent experiments (±SEM). **c,** VSVΔG(ACE2)pp were incubated over a period of 82 hours at the indicated temperature and transferred on HEK293T cells expressing SARS-CoV-2 Spike. Remianing fusogenic activity of the particles was automatically quantified by counting GFP+ particles (n=3, ±SEM) **d,** Automated quantification of GFP+ CaCo-2 cells that were infected with VSVΔG(Spike)pp, pretreatet with recombinant human soluble ACE2 or VSVΔG(ACE2)pp and PBS. Lines represent the mean of three independent experiments (±SEM).

**
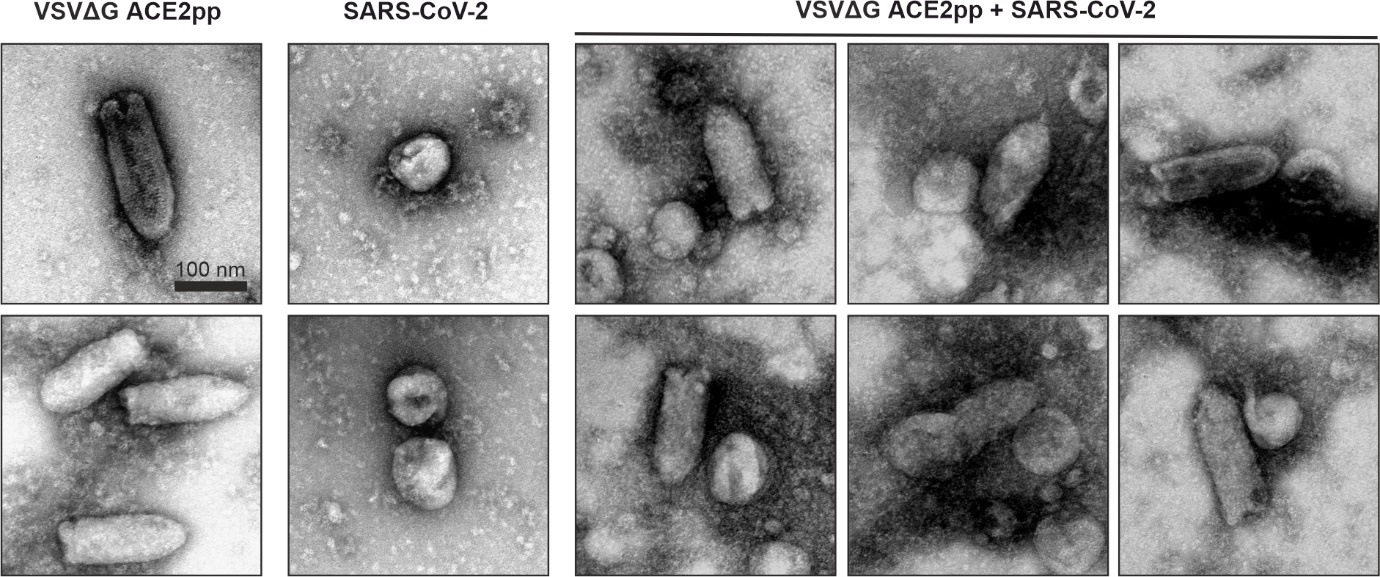
**

**Supplementary Fig. S5: VSVΔG(GFP)ACE2 pseudo-particles cluster with SARS-CoV-2 virions.** Negative staining of VSVΔG(GFP)ACE2pp alone (left), SARS-CoV-2 virions alone (middle) and both combined (right). Supernatants were left to adhere on formvar coated electron microscopy grids, negative stained with 2% uranyl acetate in aqua bidest and analyzed in a transmission electron microscope. All images show the same magnification.

**
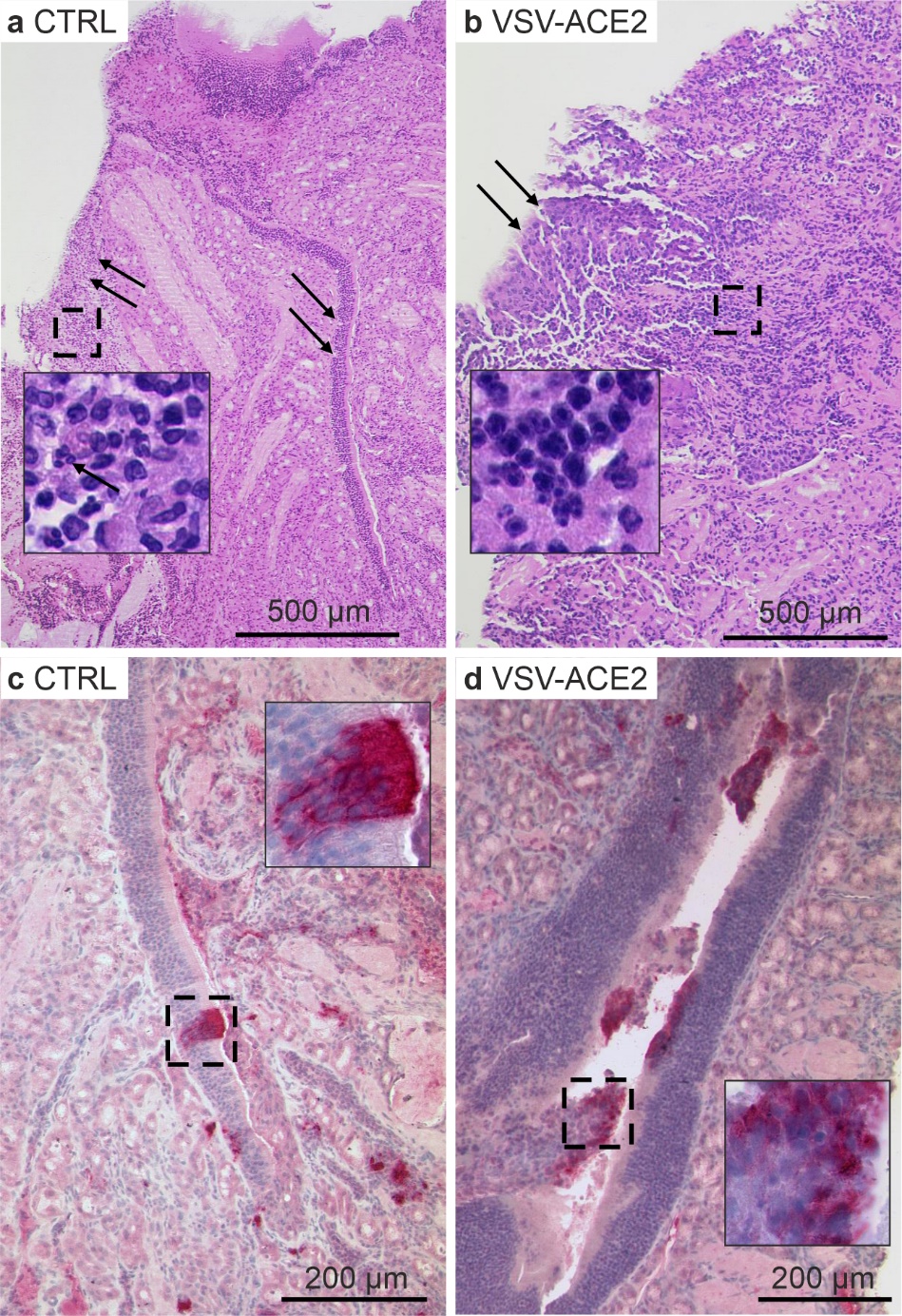
**

**Supplementary Fig. S6: Morphological spectrum and detection of spike protein by immunohistochemistry of SARS-CoV-2 infection in nasal mucosa.** **a,** Nasal mucosa shows a strong, subepithelial lymphoid infiltrate (two arrows). Insert shows lymphoid cells with some intermingled granulocytes (single arrow). **b,** In treated animals the infiltrate persists beneath the epithelium (two arrows; square marks corresponding insert with magnified lymphoid cells). **c,** Spike protein is detected in a sector-like fashion in the ciliated epithelium of the nasal mucosa (see square and insert with magnification). **d,** In treated animals spike protein persists in the nasal mucosal and intensity is close to identical as seen in control animals.

**
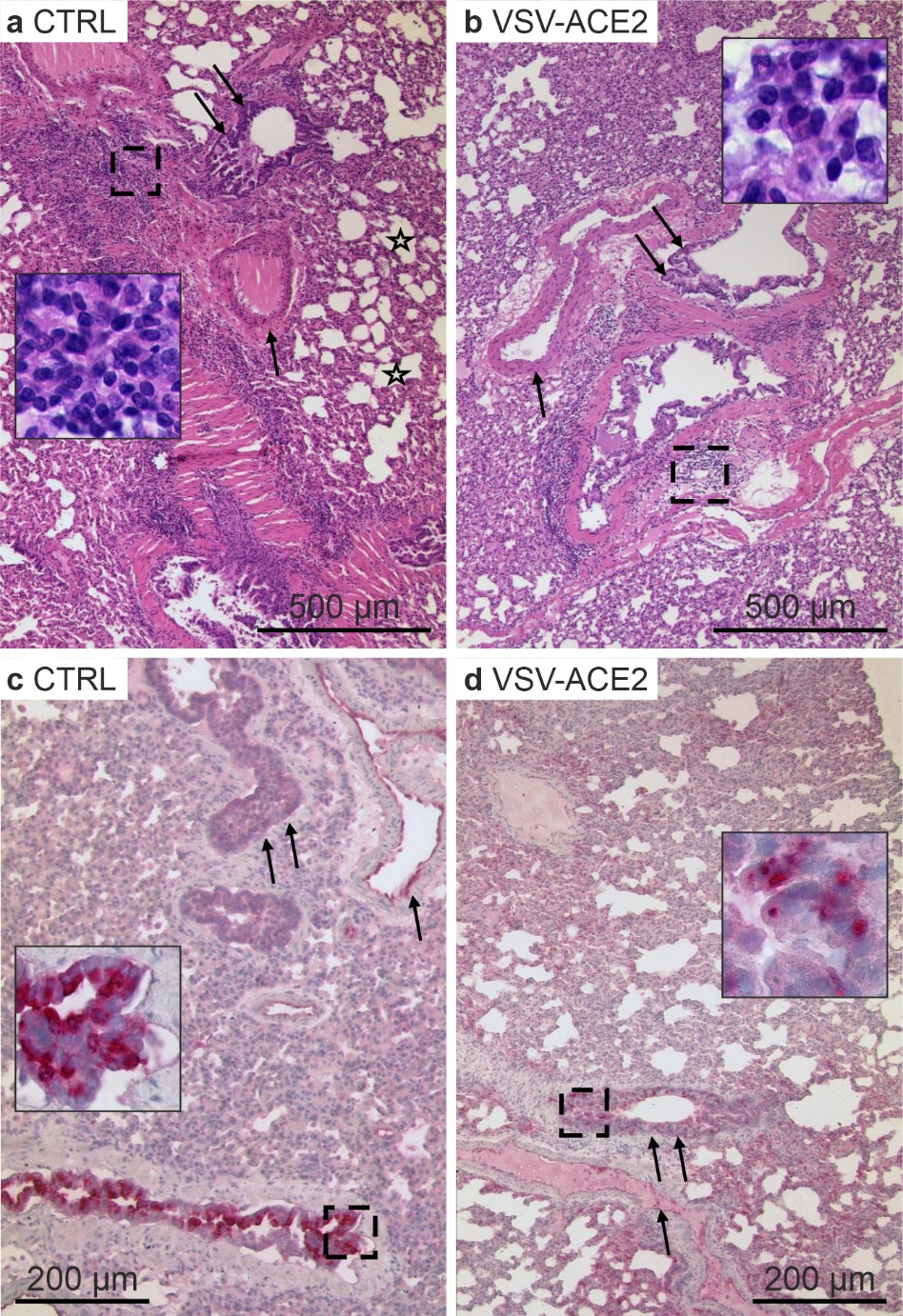
**

**Supplementary Fig. S7: Morphological spectrum and detection of spike protein by immunohistochemistry of SARS-CoV-2 infection in lung in control and VSVΔG-ACE2. a,** In control tissue a lymphoid infiltrate is seen around bronchioles (see two arrows) while alveolar spaces show no infiltrates (marked by two asterisks; magnification of lymphoid cells corresponds to area marked by a square with dashed lines). A vessel is marked by a single arrow. **b,** In the treated animals the peribronchiolar infiltrate persists although in a lower number of lymphocytes (two arrows mark bronchiolar epithelium; insert corresponds to high magnification of lymphoid infiltrates; single arrow marks a vessel). **c,** Immunohistochemistry shows a sector-like positivity of spike protein in bronchiolar epithelium stained in red; spike-negative epithelium is marked by two arrows; endothelial cells of a vessel are spike protein-positive (one arrow). Insert shows magnification of positive bronchiolar cells marked by square with dashed lines. **d,** Treated animals show a reduced, spotty like spike protein expression in bronchiolar epithelium (two arrows); endothelial cells are stained weaker compared to control (one arrow). Insert shows magnification of area marked by square with dashed lines.

3. Captions for movies

**Movie S1 and S2. Infection of Spike-expressing cells by VSVΔG-ACE2 particles.** The movies show two representative examples. VSVΔG(ACE2)pp were transferred onto a heterogeneous population of WT and SARS-CoV-2 S-expressing HEK293T cells. The cells were monitored for Spike (red) expression and VSVΔG (GFP) infection over a period of 10 hours in 30-minute intervals.

**4. Supplementary References**

1. Zech, F. et al. Spike residue 403 affects binding of coronavirus spikes to human ACE2. *Nat Commun.* **12**, 6855 (2021).

2. Hayn, M. et al. Systematic functional analysis of SARS-CoV-2 proteins uncovers viral innate immune antagonists and remaining vulnerabilities. *Cell Rep.* **35**, 109126 (2021).

3. Fornes, O. et al. A multimorphic mutation in IRF4 causes human autosomal dominant combined immunodeficiency. *Sci Immunol.* **8**, eade7953 (2023).

4. Weil, T. et al. Advanced Molecular Tweezers with Lipid Anchors against SARS-CoV-2 and Other Respiratory Viruses. *J Am Chem Soc.* **2,** 2187-2202 (2022).
